# An explicit time-domain method for non-stationary random analysis of nonlinear frame structures with plastic hinges

**DOI:** 10.1038/s41598-022-19856-4

**Published:** 2022-09-25

**Authors:** Huan Huang, Yuyu Li, Wenxiong Li, Guihe Tang, Yanmei Lv

**Affiliations:** College of Water Conservancy and Civil Engineering, South China Agricultural University, Guangzhou, 510642 China

**Keywords:** Civil engineering, Computational science

## Abstract

In this study, a novel approach for random vibration analysis of nonlinear frame structures under seismic random excitations is developed. The explicit time-domain method is improved in this approach by integrating the plastic hinge model, which can simulate the nonlinear behaviors caused by material property changes. Specifically, the hysteretic system’s equation of motion is constructed using auxiliary differential equations that govern the plastic rotational displacements and their corresponding hysteretic displacements. Additionally, by introducing the concept of equivalent excitations, an explicit iteration scheme for solving the equation of the hysteretic system is developed, in which the auxiliary differential equations are solved under the assumption that the plastic rotational velocity changes linearly with time between two adjacent time instants. Finally, by combining the Monte Carlo simulation method and the proposed explicit time-domain method, the non-stationary random responses of nonlinear frame structures can be obtained. As illustrated by numerical examples, the proposed method achieves satisfactory solution accuracy and efficiency when applied to nonlinear frame structures with plastic hinges. Moreover, the proposed iterative method resolves equations involving displacements describing the frame’s global state, plastic rotational displacements, and corresponding hysteretic parameters, introducing a novel concept for solving problems with nonlinear coupled variables of multiple types.

## Introduction

The frame structure is one of the most prevalently used structural variety in industrial and civil constructions. Under random excitations, such as seismic and wind excitations, frame structures can transition from a linear to an inelastic state^1,2^. In such a case, a certain number of plastic hinges may form when the frame structures are subjected to heavy loads, and the energy dissipation of the frame structures can be observed in the hysteretic curves of the plastic hinges. The researchers’ plastic hinges are primarily classified into two types: concentrated plastic hinge^1,3–6^, in which the plastic hinges are simulated by rotational springs at the ends of a linear-elastic beam element, as well as distributed plastic hinges^7–11^, in which the plastic hinge can occur anywhere along the beam element. While distributed plastic hinges are capable of handling more complex dynamic problems than concentrated plastic hinges, the concentrated plastic hinge is more prevalent among researchers owing to its pragmatic simplicity^1,3^.

Numerous methods for analyzing random vibrations in nonlinear systems subjected to random excitations already exist, for instance, the Fokker–Planck-Kolmogorov equation method^12^, the stochastic average method^13,14^, the moment equation method^15^, the equivalent linearization method^16–20^, the probability density evolution method^21–23^ and the Monte Carlo simulation (MCS) method^24,25^. It should be noted that, with the exception of the equivalent linearization method, the probability density evolution method, and the MCS method, the applications of the first three methods are limited by the scale of nonlinear systems and the type of random excitations. The main idea of equivalent linearization method is to substitute the original nonlinear system with an equivalent linear system. The principal drawback of this method is that the response may deviate from its true value as the system’s nonlinearity increases due to the method’s assumption of Gaussian distribution for the responses^26^. The probability density evolution method has been applied to a variety of scenarios, including the modelling of dynamic excitations of structures and the global reliability evaluation of complex structural systems. However, the computational cost exponentially increases as the number of random parameters grows. The MCS method is the only tractable approach that is applicable to a wide variety of nonlinear systems and to all cases of random excitation. Nonetheless, the MCS method is challenging to implement in practice due to the high computational cost associated with sample tests. Considering that deterministic dynamic analysis is required for each sample test, the key of MCS method is to enhance the computational efficiency of each single deterministic dynamic analysis. To address this issue, the explicit time-domain method (ETDM)^2,19,27–29^ is developed which significantly reduces the running time of MCS by enabling rapid reanalysis of each deterministic structure under different excitations.

The development of ETDM with a plastic hinge model is pivotal for random vibration analysis of nonlinear frame structures. It is capable of not only describing the nonlinear factors in a structure with a simple model, but also meeting the calculation efficiency requirements of engineering applications. With the inclusion of the plastic hinge model, the structure’s motion equation will be altered from the conventional one. In general, it has three distinct state variables: displacements describing the frame’s global state, additional plastic rotational displacements describing the deformation of the plastic hinge, and the Bouc-Wen model’s hysteretic parameters^1^. The coupling relationship between the three distinct state variables complicates the problem. The primary challenge in developing ETDM is to implement an explicit iterative solution based on the relation between the three state variables. The purpose of this paper is to propose a new framework for the explicit time-domain method that incorporates the effects of plastic hinges. The hysteretic system’s equation of motion is constructed in this framework by employing auxiliary differential equations that govern the plastic rotational displacements and corresponding hysteretic parameters. Then, using the assumption that the plastic rotational velocity changes linearly with time between two adjacent time instants, a double-layer iteration strategy is developed to solve the equation of the hysteretic system by resolving the auxiliary differential equations. The new framework markedly enhances the computational efficiency of each deterministic dynamic analysis, allowing for rapid implementation of the MCS method for the random vibration analysis of nonlinear frame structures with plastic hinges subjected to non-stationary random excitations. Numerical examples are provided to demonstrate the proposed method’s validity and effectiveness.

### Beam element with plastic hinge

**Figure 1:**
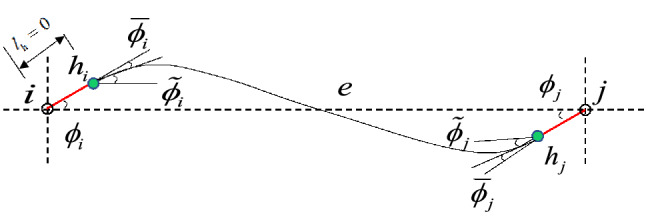
Physical model of a beam element with plastic hinges.

It is assumed that the plastic hinges $h_{i}$ and $h_{j}$ occur at the two ends of a beam element under dynamic loads, as is shown in Fig. 1. The mass of the beam is evenly distributed to node $i$ and node $j$ of the element. Thus, the part between the plastic hinges $h_{i}$ and $h_{j}$ can be regarded as an elastic element. The length of the plastic hinges is overlooked, namely, $l_{{\text{h}}} = 0$. As for a plane beam element, there generally exist three forces at each node of the beam, which includes the axial force $N_{k} \left( {k = i,j} \right)$, the shear force $S_{k} \left( {k = i,j} \right)$, and the bending moment $M_{k} \left( {k = i,j} \right)$. The corresponding six displacement components of the two nodes can be denoted by $u_{i}$, $v_{i}$, $\phi_{i}$ and $u_{j}$, $v_{j}$, $\phi_{j}$, respectively. $\overline{\phi }_{i}$ and $\overline{\phi }_{j}$ represent the rotational displacements of the plastic hinges $h_{i}$ and $h_{j}$ relative to $\phi_{i}$ and $\phi_{j}$, respectively. The rotational displacements of the ends of the middle elastic element ($\tilde{\phi }_{i}$ and $\tilde{\phi }_{j}$) are expressed as^1^

1 $$\left\{ \begin{gathered} \tilde{\phi }_{i} = \phi_{i} - \overline{\phi }_{i} \hfill \\ \tilde{\phi }_{j} = \phi_{j} - \overline{\phi }_{j} \hfill \\ \end{gathered} \right.$$

The element force vector and element displacement vectors are defined as.

2 $$\left\{ \begin{gathered} {\varvec{F}}_{e} = [N_{i} \, S_{i} \, M_{i} \, N_{j} \, S_{j} \, M_{j} ]^{{\text{T}}} \hfill \\ {\hat{\varvec{U}}}_{e} = [u_{i} \, v_{i} \, \phi_{i} \, u_{j} \, u_{j} \, \phi_{j} ]^{{\text{T}}} \hfill \\ {\overline{\varvec{U}}}_{e} = [\overline{\phi }_{i} \, \overline{\phi }_{j} ]^{{\text{T}}} \hfill \\ \end{gathered} \right.$$

where ${\varvec{F}}_{e}$ is the force vector containing the six forces of two ends of beam element $e$; ${\hat{\varvec{U}}}_{e}$ is the nodal displacement vector corresponding to beam element $e$ in the local coordinate; ${\overline{\varvec{U}}}_{e}$ is the plastic displacement vector corresponding to the angular displacements of beam element $e$ in the local coordinate. Then, the nodal forces for the elastic element between the two plastic hinges $h_{i}$ and $h_{j}$ satisfy the following equilibrium condition.

3 $${\varvec{F}}_{e} = {\varvec{K}}_{e} {\hat{\varvec{U}}}_{e} - {\varvec{K}}_{e}^{{\text{h}}} {\overline{\varvec{U}}}_{e}$$

where ${\varvec{K}}_{e}$ indicates the elastic stiffness matrix of the beam element and ${\varvec{K}}_{e}^{{\text{h}}}$ refers to the matrix corresponding to the $\overline{\phi }_{i}$ and $\overline{\phi }_{j}$, which can be expressed as^1^

4 $${\varvec{K}}_{e} = \frac{1}{{l^{3} }}\left[ {\begin{array}{*{20}c} {EAl^{2} } & 0 & 0 & { - EAl^{2} } & 0 & 0 \\ 0 & {12EI} & {6EIl} & 0 & { - 12EI} & {6EIl} \\ 0 & {6EIl} & {4EIl^{2} } & 0 & { - 6EIl} & {2EIl^{2} } \\ { - EAl^{2} } & 0 & 0 & {EAl^{2} } & 0 & 0 \\ 0 & { - 12EI} & { - 6EIl} & 0 & {12EI} & { - 6EIl} \\ 0 & {6EIl} & {2EIl^{2} } & 0 & { - 6EIl} & {4EIl^{2} } \\ \end{array} } \right],\,{\varvec{K}}_{e}^{h} = \frac{1}{{l^{3} }}\left[ {\begin{array}{*{20}c} 0 & 0 \\ {6EIl} & {6EIl} \\ {4EIl^{2} } & {2EIl^{2} } \\ 0 & 0 \\ { - 6EIl} & { - 6EIl} \\ {2EIl^{2} } & {4EIl^{2} } \\ \end{array} } \right]$$

where $E$ is the elastic modulus of beam element $e$; $A$ is the cross-sectional area of beam element $e$; $I$ is the cross-sectional moment of inertia for beam element $e$; $l$ is the length of beam element $e$.

The bending moments of the plastic hinges can be written as^1^

5 $$\left\{ \begin{gathered} P_{i} = \alpha_{i} k_{i} \overline{\phi }_{i} + (1 - \alpha_{i} )k_{i} z_{i} \hfill \\ P_{j} = \alpha_{j} k_{j} \overline{\phi }_{j} + (1 - \alpha_{j} )k_{j} z_{j} \hfill \\ \end{gathered} \right.$$

where $z_{i}$ and $z_{j}$ denote the hysteretic displacements of the rotational displacement for the plastic hinges; $k_{i}$ and $k_{j}$ represent the rotational stiffness of the plastic hinges; $\alpha_{i}$ and $\alpha_{j}$ represent the reduction ratio of the rotational stiffness of the plastic hinges, respectively; Eq. (5) can be written in the matrix form as.

6 $${\varvec{P}}_{e} = {\varvec{K}}_{p} {\overline{\varvec{U}}}_{e} + {\varvec{K}}_{r} {\varvec{Z}}_{e}$$

where ${\varvec{P}}_{e}$ denotes the bending moment vector of the plastic hinges expressed as.

7 $${\varvec{P}}_{e} { = }\left\{ {\begin{array}{*{20}c} {P_{i} } \\ {P_{j} } \\ \end{array} } \right\}$$

${\varvec{K}}_{p}$ and ${\varvec{K}}_{r}$ in Eq. (6) are written as.

8 $${\varvec{K}}_{p} { = }\left[ {\begin{array}{*{20}c} {\alpha_{i} k_{i} } & 0 \\ 0 & {\alpha_{j} k_{j} } \\ \end{array} } \right],\,{\varvec{K}}_{r} { = }\left[ {\begin{array}{*{20}c} {(1 - \alpha_{i} )k_{i} } & 0 \\ 0 & {(1 - \alpha_{j} )k_{j} } \\ \end{array} } \right]$$

It is noted that $z_{i}$ and $z_{j}$ are governed by the following equation^30^

9 $$\left\{ \begin{gathered} \dot{z}_{i} = a_{i} \dot{\overline{\phi }}_{i} - \varphi_{i} |\dot{\overline{\phi }}_{i} |z_{i} |z_{i} |^{{\theta_{i} - 1}} - \psi_{i} \dot{\overline{\phi }}_{i} |z_{i} |^{{\theta_{i} }} \hfill \\ \dot{z}_{j} = a_{j} \dot{\overline{\phi }}_{j} - \varphi_{j} |\dot{\overline{\phi }}_{j} |z_{j} |z_{j} |^{{\theta_{j} - 1}} - \psi_{j} \dot{\overline{\phi }}_{j} |z_{j} |^{{\theta_{j} }} \hfill \\ \end{gathered} \right.$$

where $a_{i}$, $a_{j}$, $\varphi_{i}$, $\varphi_{j}$, $\psi_{i}$, $\psi_{j}$, $\theta_{i}$ and $\theta_{j}$ are the parameters of the corresponding hysteretic displacements of the plastic hinges. Eq. (9) can be rearranged as.

10 $${\dot{\varvec{Z}}}_{e} = {\varvec{A}}_{e} {{\dot{\overline{\varvec{U}}}}}_{e} - {{\varvec{\Phi}}}_{e} {\varvec{B}}_{e} ({{\dot{\overline{\varvec{U}}}}}_{e} , {\varvec{Z}}_{e} ) - {{\varvec{\Psi}}}_{e} {\varvec{D}}_{e} ({{\dot{\overline{\varvec{U}}}}}_{e} {, }{\varvec{Z}}_{e} )$$

where

11 $$\left\{ \begin{gathered} {\dot{\varvec{Z}}}_{e} = \left\{ {\begin{array}{*{20}c} {\dot{z}_{i} } \\ {\dot{z}_{j} } \\ \end{array} } \right\},{\varvec{A}}_{e} = \left[ {\begin{array}{*{20}c} {a_{i} } & 0 \\ 0 & {a_{j} } \\ \end{array} } \right],{{\varvec{\Phi}}}_{e} = \left[ {\begin{array}{*{20}c} {\varphi_{i} } & 0 \\ 0 & {\varphi_{j} } \\ \end{array} } \right],{{\varvec{\Psi}}}_{e} = \left[ {\begin{array}{*{20}c} {\psi_{i} } & 0 \\ 0 & {\psi_{j} } \\ \end{array} } \right] \hfill \\ {\varvec{B}}_{e} ({{\dot{\overline{\varvec{U}}}}}_{e} {, }{\varvec{Z}}_{e} ) = \left\{ {\begin{array}{*{20}c} {|\dot{\overline{\phi }}_{i} |z_{i} |z_{i} |^{{\theta_{i} - 1}} } \\ {|\dot{\overline{\phi }}_{j} |z_{j} |z_{j} |^{{\theta_{j} - 1}} } \\ \end{array} } \right\},{\varvec{D}}_{e} ({{\dot{\overline{\varvec{U}}}}}_{e} {, }{\varvec{Z}}_{e} ) = \left\{ {\begin{array}{*{20}c} {\dot{\overline{\phi }}_{i} |z_{i} |^{{\theta_{i} }} } \\ {\dot{\overline{\phi }}_{j} |z_{j} |^{{\theta_{j} }} } \\ \end{array} } \right\} \hfill \\ \end{gathered} \right.$$

In addition, the bending moments of the plastic hinges and the elastic element satisfy the following equilibrium equation.

12 $$\left\{ {\begin{array}{*{20}c} {P_{i} } \\ {P_{j} } \\ \end{array} } \right\} = \left\{ {\begin{array}{*{20}c} {M_{i} } \\ {M_{j} } \\ \end{array} } \right\} = {\varvec{LF}}_{e}$$

where ${\varvec{L}}$ is the position matrix expressed as.

13 $${\varvec{L}} = \left[ {\begin{array}{*{20}c} 0 & 0 & 1 & 0 & 0 & 0 \\ 0 & 0 & 0 & 0 & 0 & 1 \\ \end{array} } \right]$$

By substituting Eq. (3) and Eq. (6) into Eq. (12), one can obtain.

14 $${\varvec{LK}}_{e} ({\hat{\varvec{U}}}_{e} - {\varvec{L}}^{{\text{T}}} {\overline{\varvec{U}}}_{e} ) - ({\varvec{K}}_{P} {\overline{\varvec{U}}}_{e} + {\varvec{K}}_{r}\, {\varvec{Z}}_{e} ) = \varvec{0}$$

Considering the relationship of the nodal displacements in the local coordinate system and that in the global coordinate system, one can obtain.

15 $${\hat{\varvec{U}}}_{e} = {\varvec{TU}}_{e}$$

where ${\varvec{T}}$ is the transformation matrix of coordinates; ${\varvec{U}}_{e}$ is the nodal displacement of the beam element $e$ in the global coordinate system. Substituting Eq. (15) into Eq. (14) yields.

16 $${\varvec{Z}}_{e} = {\varvec{R}}_{e,1} {\varvec{U}}_{e} + {\varvec{R}}_{e,2} {\overline{\varvec{U}}}_{e}$$

where

17 $$\left\{ \begin{gathered} {\varvec{R}}_{e,1} { = }{\varvec{K}}_{r}^{ - 1} {\varvec{LK}}_{e} {\varvec{T}} \hfill \\ {\varvec{R}}_{e,2} = - {\varvec{K}}_{r}^{ - 1} ({\varvec{LK}}_{e} {\varvec{L}}^{{\text{T}}} + {\varvec{K}}_{P} ) \hfill \\ \end{gathered} \right.$$

Taking the derivative of Eq. (16) with respect to time coordinate $t$, one can obtain.

18 $${\dot{\varvec{Z}}}_{e} = {\varvec{R}}_{e,1} {\dot{\varvec{U}}}_{e} + {\varvec{R}}_{e,2} {{\dot{\overline{\varvec{U}}}}}_{e}$$

Based on Eq. (10) and Eq. (18), one can obtain.

19 $${{\dot{\overline{\varvec{U}}}}}_{e} = {\varvec{R}}_{e,3} {\dot{\varvec{U}}}_{e} + {\varvec{R}}_{e,4} {\varvec{B}}_{e} ({{\dot{\overline{\varvec{U}}}}}_{e} {, }{\varvec{Z}}_{e} ) + {\varvec{R}}_{e,5} {\varvec{D}}_{e} ({{\dot{\overline{\varvec{U}}}}}_{e} {, }{\varvec{Z}}_{e} )$$

where

20 $$\left\{ \begin{gathered} {\varvec{R}}_{e,3} = - ({\varvec{R}}_{e,2} - {\varvec{A}}_{e} )^{ - 1} {\varvec{R}}_{e,1} \hfill \\ {\varvec{R}}_{e,4} = - ({\varvec{R}}_{e,2} - {\varvec{A}}_{e} )^{ - 1} {{\varvec{\Phi}}}_{e} \hfill \\ {\varvec{R}}_{e,5} = - ({\varvec{R}}_{e,2} - {\varvec{A}}_{e} )^{ - 1} {{\varvec{\Psi}}}_{e} \hfill \\ \end{gathered} \right.$$

Then, the auxiliary differential equation of rotational displacements for the plastic hinges can be obtained.

### Nonlinear system with plastic hinge elements

As for the whole nonlinear structure with plastic hinge elements, when the assembly of all elements is complete, the equation of motion for the whole system with *n* degrees of freedom (DOFs) can be expressed as^1^

21 $${\varvec{M}}{\ddot{\varvec{U}}} + {\varvec{C}}{\dot{\varvec{U}}} + {\varvec{KU}} + {\varvec{K}}^{{\text{h}}} {\overline{\varvec{U}}} = {\boldsymbol{\Theta}} {\varvec{F}}(t)$$

where ${\varvec{M}}$ and ${\varvec{C}}$ are the mass matrix and the damping matrix, respectively; ${\varvec{K}}$ is the initial linear stiffness matrix; ${\varvec{K}}^{{\text{h}}}$ is the stiffness matrix corresponding to the rotational displacements of all plastic hinges; ${\varvec{U}}$, ${\dot{\varvec{U}}}$ and ${{\ddot{\varvec{U}}}}$ are the displacement vector, the velocity vector and the acceleration vector in the global coordinate system, respectively; ${\overline{\varvec{U}}}$ is the rotational displacement vectors of the plastic hinges in the global coordinate system; ${{\varvec{\Theta}}}$ is an $n \times m$ position matrix with $m$ the index for each external load; ${\varvec{F}}(t){ = }[{\varvec{F}}_{1} (t) \, {\varvec{F}}_{2} (t) \, \cdots \, {\varvec{F}}_{m} (t)]^{{\text{T}}}$ is the non-stationary load vector.

In addition, Eq. (16) and Eq. (19) are assembled in the global coordinate system, which are written as.

22 $${\varvec{Z}} = {\varvec{R}}_{1} {\varvec{U}}^{*} + {\varvec{R}}_{2} {\overline{\varvec{U}}}$$

23 $${\dot{\overline{\varvec{U}}}} = {\varvec{R}}_{3} {\dot{\varvec{U}}}^{*} + {\varvec{R}}_{4} {\varvec{B}}({\dot{\overline{\varvec{U}}}}, {\varvec{Z}}) + {\varvec{R}}_{5} {\varvec{D}}({\dot{\overline{\varvec{U}}}}, {\varvec{Z}})$$

where ${\varvec{R}}_{1}$, ${\varvec{R}}_{2}$, ${\varvec{R}}_{3}$, ${\varvec{R}}_{4}$ and ${\varvec{R}}_{5}$ are assembled through ${\varvec{R}}_{e,1}$, ${\varvec{R}}_{e,2}$, ${\varvec{R}}_{e,3}$, ${\varvec{R}}_{e,4}$ and ${\varvec{R}}_{e,5}$, respectively; ${\varvec{U}}^{*}$ is the nodal displacements connected with the plastic hinges, which is extracted from ${\varvec{U}}$; ${\dot{\varvec{U}}}^{*}$ is the first derivative of ${\varvec{U}}^{*}$.

## Implementation of the explicit iteration method

In order to solve Eqs. (21) and (23), the three sets of coupled variables are needed to be determined simultaneously. In this study, the decoupling of variables stated in Ref.^2^ is used to cope with the three sets of variables, respectively.

### Recursion formula for responses of quasi-linear motion equations

Eq. (21) can be rewritten as the following second-order quasi-linear equilibrium equation of motion for the nodal displacement vector by moving the term ${\varvec{K}}^{{\text{h}}} {\overline{\varvec{U}}}$ to the right-hand side of the equation.

24 $${\varvec{M}}{\ddot{\varvec{U}}} + {\varvec{C}}{\dot{\varvec{U}}} + {\varvec{KU}} = {\tilde{\varvec{F}}}(t)$$

where ${\tilde{\varvec{F}}}(t)$ can be regarded as an equivalent dynamic loading vector including the rotational displacements of the plastic hinges and expressed as.

25 $${\tilde{\varvec{F}}}(t){ = }{\boldsymbol{\Theta}} {\varvec{F}}(t) - {\varvec{K}}^{{\text{h}}} {\overline{\varvec{U}}}$$

Eq. (24) can be solved by the Newmark-*β* method. For the Newmark-*β* method, the following assumptions are used as follows^31,32^

26 $$\begin{array}{*{20}c} {{\dot{\varvec{U}}}_{i} = {\dot{\varvec{U}}}_{i - 1} + [(1 - \gamma ){{\ddot{\varvec{U}}}}_{i - 1} + \gamma {{\ddot{\varvec{U}}}}_{i} ]\Delta t} & {\left( {i = 0,1,2, \cdots ,l} \right)} \\ \end{array}$$

27 $$\begin{array}{*{20}c} {{\varvec{U}}_{i} = {\varvec{U}}_{i - 1} + {\dot{\varvec{U}}}_{i - 1} \Delta t + \frac{1}{2}[(1 - 2\beta ){{\ddot{\varvec{U}}}}_{i - 1} + 2\beta {{\ddot{\varvec{U}}}}_{i} ]\Delta t^{2} } & {\left( {i = 0,1,2, \cdots ,l} \right)} \\ \end{array}$$

where $\gamma$ and $\beta$ are two parameters used to control the Newmark-*β* integration stability; $l = \tau /\Delta t$ with $\tau$ and $\Delta t$ being the total time and the time step, respectively; The subscripts “$i - 1$” and “$i$” denote $t_{i - 1} = (i - 1)\Delta t$ and $t_{i} = i\Delta t$, respectively. In this study, $\gamma = 0.5$ and $\beta = 0.25$ are used and the Newmark-*β* method will be unconditionally stable. Based on Eqs. (26) and (27), one can obtain the acceleration and the velocity at time instant $t_{i}$ and they can be expressed as

28 $$\begin{array}{*{20}c} {{{\ddot{\varvec{U}}}}_{i} = a_{0} ({\varvec{U}}_{i} - {\varvec{U}}_{i - 1} ) - a_{1} {\dot{\varvec{U}}}_{i - 1} - a_{2} {{\ddot{\varvec{U}}}}_{i - 1} } & {\left( {i = 0,1,2, \cdots ,l} \right)} \\ \end{array}$$

29 $$\begin{array}{*{20}c} {{\dot{\varvec{U}}}_{i} = a_{3} ({\varvec{U}}_{i} - {\varvec{U}}_{i - 1} ) - a_{4} {\dot{\varvec{U}}}_{i - 1} - a_{5} {{\ddot{\varvec{U}}}}_{i - 1} } & {\left( {i = 0,1,2, \cdots ,l} \right)} \\ \end{array}$$

where

30 $$\left\{ \begin{gathered} a_{0} = \frac{1}{{\beta \Delta t^{2} }}, \, a_{1} = \frac{1}{\beta \Delta t}, \, a_{2} = \frac{1}{2\beta } - 1 \hfill \\ a_{3} = \frac{\gamma }{\beta \Delta t}, \, a_{4} = \frac{\gamma }{\beta } - 1, \, a_{5} = \frac{\Delta t}{2}(\frac{\gamma }{\beta } - 2) \hfill \\ \end{gathered} \right.$$

The equation of motion for the quasi-linear system at time instant $t_{i}$ can be written as.

31 $${\varvec{M\ddot{U}}}_{i} + {\varvec{C\dot{U}}}_{i} + {\varvec{KU}}_{i} = {\tilde{\varvec{F}}}_{i}$$

By substituting Eqs. (28) and (29) into Eq. (31), it yields

32 $${\varvec{U}}_{i} = {\hat{\varvec{K}}}^{ - 1} {\hat{\varvec{F}}}_{i}$$

where

33 $${\hat{\varvec{K}}} = {\varvec{K}} + a_{0} {\varvec{M}} + a_{3} {\varvec{C}}$$

34 $${\hat{\varvec{F}}}_{i} = {\tilde{\varvec{F}}}_{i} + {\varvec{M}}(a_{0} {\varvec{U}}_{i - 1} + a_{1} {\dot{\varvec{U}}}_{i - 1} + a_{2} {\ddot{\varvec{U}}}_{i - 1} ) + {\varvec{C}}(a_{3} {\varvec{U}}_{i - 1} + a_{4} {\dot{\varvec{U}}}_{i - 1} + a_{5} {\ddot{\varvec{U}}}_{i - 1} )$$

In addition, based on Eq. (31), the acceleration vector at time instant $t_{i}$ can also expressed as.

35 $${\ddot{\varvec{U}}}_{i} = {\varvec{M}}_{{}}^{ - 1} [{\tilde{\varvec{F}}}_{i} - {\varvec{C\dot{U}}}_{i} - {\varvec{KU}}_{i} ]$$

Analogously, one has.

36 $${\ddot{\varvec{U}}}_{i - 1} = {\varvec{M}}_{{}}^{ - 1} [{\tilde{\varvec{F}}}_{i - 1} - {\varvec{C\dot{U}}}_{i - 1} - {\varvec{KU}}_{i - 1} ]$$

By substituting Eq. (36) into Eq. (34), it yields

37 $${\varvec{U}}_{i} = {\varvec{H}}_{11} {\varvec{U}}_{i - 1} + {\varvec{H}}_{12} {\dot{\varvec{U}}}_{i - 1} + {\varvec{R}}_{1} {\hat{\varvec{F}}}_{i - 1} + {\varvec{R}}_{2} {\hat{\varvec{F}}}_{i}$$

where

38 $$\left\{ \begin{gathered} {\varvec{H}}_{11} = {\hat{\varvec{K}}}^{ - 1} ({\varvec{S}}_{1} - {\varvec{S}}_{3} {\varvec{M}}^{ - 1} {\varvec{K}}), \, {\varvec{H}}_{12} = {\hat{\varvec{K}}}^{ - 1} [{\varvec{S}}_{2} - {\varvec{S}}_{3} {\varvec{M}}^{ - 1} {\varvec{C}}] \hfill \\ {\varvec{R}}_{1} = {\hat{\varvec{K}}}^{ - 1} {\varvec{S}}_{3} {\varvec{M}}^{ - 1} , \, {\varvec{R}}_{2} = {\hat{\varvec{K}}}^{ - 1} \hfill \\ {\varvec{S}}_{1} = a_{0} {\varvec{M}} + a_{3} {\varvec{C}}, \, {\varvec{S}}_{2} = a_{1} {\varvec{M}} + a_{4} {\varvec{C}}, \, {\varvec{S}}_{3} = a_{2} {\varvec{M}} + a_{5} {\varvec{C}} \hfill \\ \end{gathered} \right.$$

By substituting Eq. (37) into (29) and considering Eq. (36), it yields

39 $${\dot{\varvec{U}}}_{i} = {\varvec{H}}_{21} {\varvec{U}}_{i - 1} + {\varvec{H}}_{22} {\dot{\varvec{U}}}_{i - 1} + {\varvec{R}}_{3} {\hat{\varvec{F}}}_{i - 1} + {\varvec{R}}_{4} {\hat{\varvec{F}}}_{i}$$

where

40 $$\left\{ \begin{gathered} {\varvec{H}}_{21} = a_{3} ({\varvec{H}}_{11} - \varvec{I}) + a_{5} {\varvec{M}}^{ - 1} {\varvec{K}}, \, {\varvec{H}}_{22} = a_{3} {\varvec{H}}_{12} - a_{4} \varvec{I} + a_{5} {\varvec{M}}^{ - 1} {\varvec{C}} \hfill \\ {\varvec{R}}_{3} = a_{3} {\varvec{R}}_{1} - a_{5} {\varvec{M}}^{ - 1} , \, {\varvec{R}}_{4} = a_{3} {\varvec{R}}_{2} \hfill \\ \end{gathered} \right.$$

Based on Eqs. (37) and (39), one can derive the following recursion formula

41 $$\begin{array}{*{20}c} {{\varvec{V}}_{i} = {\varvec{TV}}_{i - 1} + {\varvec{Q}}_{1} ({\boldsymbol{\Theta}} {\varvec{F}}_{i - 1} - {\varvec{K}}^{{\text{h}}} {\overline{\varvec{U}}}_{i - 1} ) + {\varvec{Q}}_{2} ({\boldsymbol{\Theta}} {\varvec{F}}_{i} - {\varvec{K}}^{{\text{h}}} {\overline{\varvec{U}}}_{i} )} & {\left( {i = 0,1,2, \cdots ,l} \right)} \\ \end{array}$$

where

42 $${\varvec{V}}_{i} = \left\{ {\begin{array}{*{20}c} {{\varvec{U}}_{i} } \\ {{\dot{\varvec{U}}}_{i} } \\ \end{array} } \right\},\,{\varvec{T}} = \left[ {\begin{array}{*{20}c} {{\varvec{H}}_{11} } & {{\varvec{H}}_{12} } \\ {{\varvec{H}}_{21} } & {{\varvec{H}}_{22} } \\ \end{array} } \right], \, {\varvec{Q}}_{1} = \left[ {\begin{array}{*{20}c} {{\varvec{R}}_{1} } \\ {{\varvec{R}}_{3} } \\ \end{array} } \right], \, {\varvec{Q}}_{2} = \left[ {\begin{array}{*{20}c} {{\varvec{R}}_{2} } \\ {{\varvec{R}}_{4} } \\ \end{array} } \right]$$

Obviously, the recursion formula in Eq. (41) can be employed to derive the nodal displacements and velocity when the load vector ${\varvec{F}}_{i}$, the rotational displacements ${\overline{\varvec{U}}}_{i}$ for the plastic hinges at the current time instant and the state vector ${\varvec{V}}_{i - 1}$, the load vector ${\varvec{F}}_{i - 1}$, the rotational displacements ${\overline{\varvec{U}}}_{i - 1}$ for the plastic hinges at the previous current time instant are given.

### Solution of hysteretic displacement and rotational displacements for plastic hinges

In fact, Eq. (23) can be expressed in another form as.

43 $${\dot{\overline{\varvec{U}}}} = {\varvec{G}}({\dot{\varvec{U}}}^{*} , \, {\dot{\overline{\varvec{U}}}}, \, {\varvec{Z}})$$

where

44 $${\varvec{G}}({\dot{\varvec{U}}}^{*} , \, {\dot{\overline{\varvec{U}}}}, \, {\varvec{Z}}) = {\varvec{R}}_{3} {\dot{\varvec{U}}}^{*} + {\varvec{R}}_{4} {\varvec{B}}({\dot{\overline{\varvec{U}}}}{, }{\varvec{Z}}) + {\varvec{R}}_{5} {\varvec{D}}({\dot{\overline{\varvec{U}}}}{, }{\varvec{Z}})$$

For a given ${\varvec{G}}({\dot{\varvec{U}}}^{*} , \, {\dot{\overline{\varvec{U}}}}, \, {\varvec{Z}})$, Eq. (43) can be treated as a simple first-order linear differential equation about ${\dot{\varvec{U}}}^{*}$, ${\dot{\overline{\varvec{U}}}} \,$ and ${\varvec{Z}}$. Assuming that ${\varvec{G}}({\dot{\varvec{U}}}^{*} , \, {\dot{\overline{\varvec{U}}}}, \, {\varvec{Z}})$ changes with time linearly between the current time instant $t_{i}$ and the previous time instant $t_{i - 1}$, the following recursion formula for ${\overline{\varvec{U}}}$ can be easily obtained.

45 $$\begin{array}{*{20}c} {{\overline{\varvec{U}}}_{i} = {\overline{\varvec{U}}}_{i - 1} + [{\varvec{G}}({\dot{\varvec{U}}}_{i - 1}^{*} , \, {\dot{\overline{\varvec{U}}}}_{i - 1} {, }{\varvec{Z}}_{i - 1} ) + {\varvec{G}}({\dot{\varvec{U}}}_{i}^{*} , \, {\dot{\overline{\varvec{U}}}}_{i} {, }{\varvec{Z}}_{i} )]\Delta t/2} & {\left( {i = 0,1,2, \cdots ,l} \right)} \\ \end{array}$$

As expressed by Eq. (22), if ${\overline{\varvec{U}}}_{i}$ and ${\varvec{U}}_{i}^{*}$ are given, the expression of ${\varvec{Z}}_{i}$ can be derived as.

46 $${\varvec{Z}}_{i} = {\varvec{R}}_{1} {\varvec{U}}_{i}^{*} + {\varvec{R}}_{2} {\overline{\varvec{U}}}_{i}$$

### Iteration algorithm

Although the recursion formulas for the state vector ${\varvec{V}}$, rotational displacement vector ${\overline{\varvec{U}}}$ and hysteretic displacement vector ${\varvec{Z}}$ has been derived (see Eqs. (41), (45) and (46)), the responses ${\varvec{V}}_{i}$, ${\overline{\varvec{U}}}_{i}$ and ${\varvec{Z}}$ at the current time instant $t_{i}$ cannot be directly obtained by using Eqs. (41) and (46) even when the responses at the previous time instant $t_{i - 1}$ are already known, because the three sets of variables are coupled. Therefore, the iteration solution process for the three sets of variables must be employed due to the consideration of interdependency for them. The iteration solution process in detail is given as follows.

1. Assign an initial value to ${\overline{\varvec{U}}}_{i}$ (Note that the initial values may be the converged result ${\overline{\varvec{U}}}_{i - 1}$ in the previous time instant);
2. Substitute ${\overline{\varvec{U}}}_{i}$ into Eqs. (41) and calculate ${\varvec{V}}_{i} = [{\varvec{U}}_{i}^{{\text{T}}} \, {\dot{\varvec{U}}}_{i}^{{\text{T}}} ]^{{\text{T}}}$;
3. This step involves the following inner loop:
  ① Assign initial values to ${\dot{\overline{\varvec{U}}}}_{i}$, ${\dot{\varvec{U}}}_{i}^{*}$ and ${\varvec{Z}}_{i}$ (the initial value of ${\dot{\varvec{U}}}_{i}^{*}$ extracted from ${\varvec{V}}_{i}$ in step (2), the converged results ${\dot{\overline{\varvec{U}}}}_{i - 1}$ and ${\varvec{Z}}_{i - 1}$ for the previous time instant are used);
  ② Substitute ${\dot{\overline{\varvec{U}}}}_{i}$, ${\dot{\varvec{U}}}_{i}^{*}$ and ${\varvec{Z}}_{i}$ into the right-hand side of Eq. (45) and calculate the new ${\overline{\varvec{U}}}_{i}$;
  ③ Substitute ${\varvec{U}}_{i}^{*}$ extracted from ${\varvec{V}}_{i}$ in step (2) and the new ${\overline{\varvec{U}}}_{i}$ in step ② into Eq. (46) and calculate the new ${\varvec{Z}}_{i}$;
  ④ Substitute ${\dot{\varvec{U}}}_{i}^{*}$, ${\dot{\overline{\varvec{U}}}}_{i}$ and the new ${\varvec{Z}}_{i}$ into Eq. (43) and calculate the new ${\dot{\overline{\varvec{U}}}}_{i}$;
  ⑤ Check whether ${\overline{\varvec{U}}}_{i}$ is converge. If not, update ${\dot{\overline{\varvec{U}}}}_{i}$, ${\dot{\varvec{U}}}_{i}^{*}$, ${\varvec{Z}}_{i}$ and repeat steps ②-④ until ${\overline{\varvec{U}}}_{i}$ is converge;

(4) Check whether ${\varvec{V}}_{i}$ is converge. If not, update ${\overline{\varvec{U}}}_{i}$ and repeat steps (2) and (3) until ${\varvec{V}}_{i}$ is converge.

**Figure 2:**
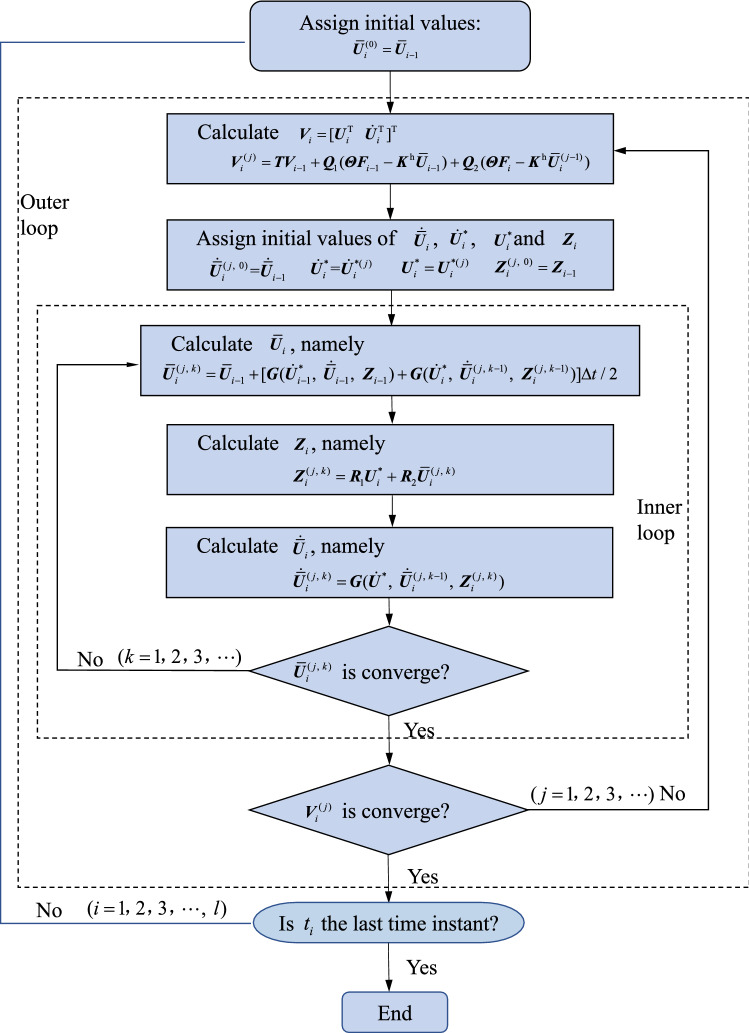
The flowchart of the solution procedure based on the proposed method.

Figure 2 displays the flow chart of the above procedure. As can be seen from Fig. 2, two iteration processes, i.e. the inner loop and the outer loop, are involved for each time instant. For the inner loop, the iteration process can be ended when ${\overline{\varvec{U}}}_{i}^{(j, \, k)}$ satisfies $\frac{{{||}{\overline{\varvec{U}}}_{i}^{(j, \, k)} - {\overline{\varvec{U}}}_{i}^{(j, \, k - 1)} {||}}}{{{||}{\overline{\varvec{U}}}_{i}^{(j, \, k)} {||}}} \le \varepsilon_{1}$ with $\varepsilon_{1}$ being an error tolerance and the symbol ${||} \bullet {||}$ denoting the Euclidean norm. For the outer loop, the iteration process can be ended when ${\varvec{V}}_{i}^{(j)}$ satisfies $\frac{{{||}{\varvec{V}}_{i}^{(j)} - {\varvec{V}}_{i}^{(j - 1)} {||}}}{{{||}{\varvec{V}}_{i}^{(j)} {||}}} \le \varepsilon_{2}$ with $\varepsilon_{2}$ being an error tolerance. When the outer loop ends, one can move on to the next time instant. It is noted that once ${\varvec{T}}$, ${\varvec{Q}}_{{1}}$, ${\varvec{Q}}_{{2}}$ and ${\varvec{R}}_{m}$ $(m = 1,{ 2, 3, 4, 5)}$ are obtained, they don’t require update throughout the solution process. Therefore, the above proposed method will be more efficient than the other numerical integration methods involving the repeated update of the effective stiffness matrix and calculating its inverse repeatedly in each time step.

## Random responses of the nonlinear systems

The MCS method is one of the main methods for random vibration analysis of nonlinear systems with the rapid development of computer technology. As for this method, a large number of excitation samples can be obtained based on its power spectrum or correlation function of random excitations through numerical simulation. One determinate nonlinear dynamic analysis is needed for each excitation sample based on the MCS method. Obviously, it is extremely time-consuming and even not practical for the MCS method based on the conventional integration scheme. In order to improve the computational efficiency of a single determinate nonlinear dynamic analysis, the explicit iteration method proposed in Sect. 4 is used in the MCS method, which will be still called the explicit iteration MCS method^2^. As depicted in Fig. 2, ${\varvec{T}}$, ${\varvec{Q}}_{{1}}$, ${\varvec{Q}}_{{2}}$ and ${\varvec{R}}_{m}$ $(m = 1,{ 2, 3, 4, 5)}$ remain the same for each time step and each excitation sample. In other words, theses matrices are calculated only once in the whole MCS process. Hence, the time elapsed by the explicit iteration MCS method will be reduced greatly.

## Numerical examples

**Figure 3:**
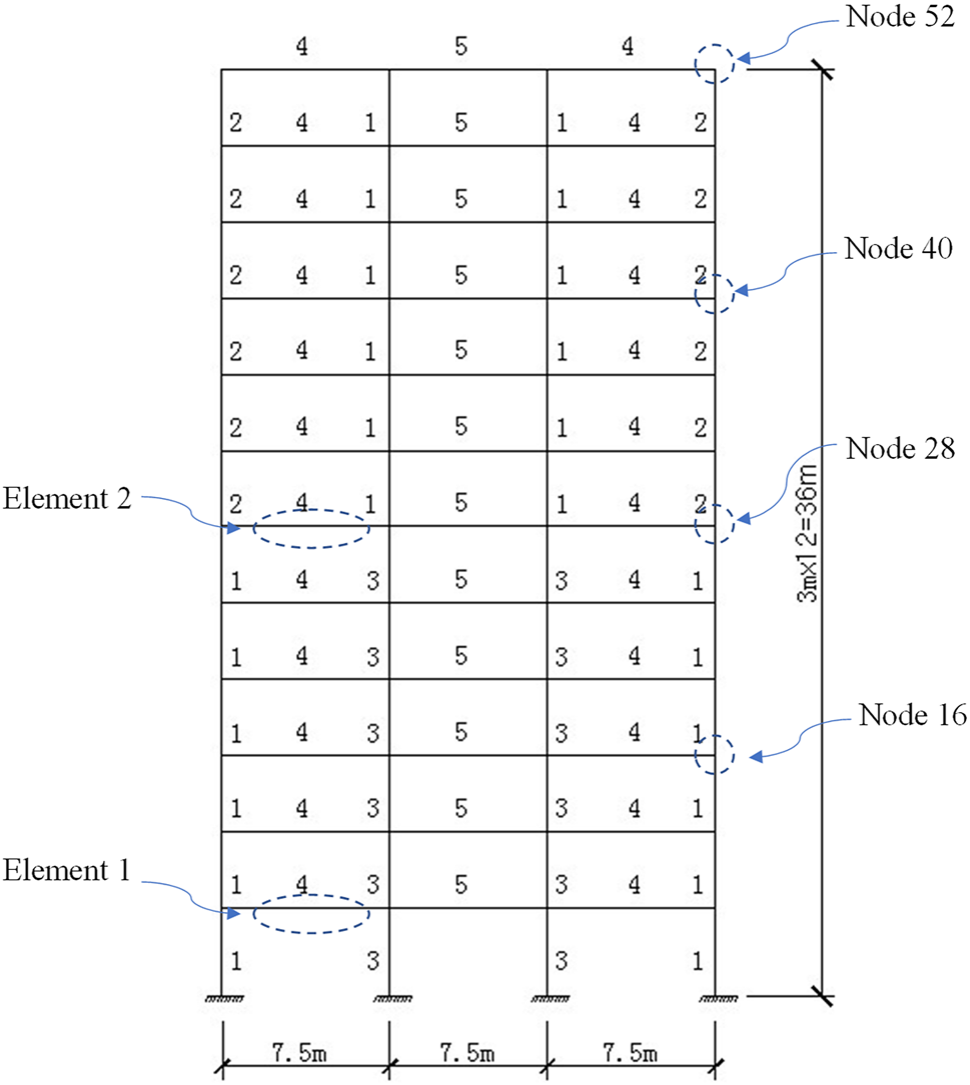
A frame structure subjected to seismic excitations.

A three-bay frame structure with twelve storeys shown in Fig. 3 is analyzed. The elastic modulus for each beam and each column is $E = 27.87\;{\text{GPa}}$ and the density is $2500\;{\text{kg/m}}^{{3}}$. The geometrical sizes for the components labeled by 1 ~ 5 are $0.6\;{\text{m}} \times 0.6\;{\text{m}}$, $0.5\;{\text{m}} \times 0.5\;{\text{m}}$, $0.7\;{\text{m}} \times 0.7\;{\text{m}}$, $0.3\;{\text{m}} \times 0.5\;{\text{m}}$ and $0.3\;{\text{m}} \times 0.5\;{\text{m}}$, respectively. The number of DOFs is $n = 144$. The lumped mass is evenly concentrated to the two ends of each component. The damping matrix for the initial linear system is defined by using Rayleigh damping model and the critical damping ration is set to 0.05 for the 1st mode and the 100th mode of the initial linear system. It is supposed that the plastic hinges only occur at the two ends of each beam. The parameters for all plastic hinges are set as $k_{i} = k_{j} = 1 \times 10^{10} \;{\text{N}} \cdot {\text{m/rad}}$, $\alpha_{i} = \alpha_{j} = 0.001$, $\varphi_{i} = \varphi_{j} = 5 \times 10^{4} \;{\text{rad}}^{ - 1}$, $\psi_{i} = \psi_{j} = 5 \times 10^{4} \;{\text{rad}}^{ - 1}$, $a_{i} = a_{j} = 1$ and $\theta_{i} = \theta_{j} = 1$.

The frame structure is subjected to non-stationary seismic excitation ${\varvec{F}}(t) = {\ddot{\varvec{X}}}_{g} (t)$, which is assumed to be a uniformly modulated random process expressed as ${\ddot{\varvec{X}}}_{g} (t) = g(t)x(t)$ with $x(t)$ being a stationary random process with zero mean. The Kanai-Tajimi spectrum^33^ is used for the power spectral density function of $x(t)$, namely.

47 $$S_{xx} (\omega ) = \frac{{\omega_{g}^{4} + 4\varsigma_{g}^{2} \omega_{g}^{2} \omega^{2} }}{{(\omega_{g}^{2} - \omega^{2} )^{2} + 4\varsigma_{g}^{2} \omega_{g}^{2} \omega^{2} }}S_{0}$$

where $\omega_{g} = 15.708\;{\text{rad/s}}$, $\varsigma_{g} = 0.6$, $S_{0} = 1.574 \times 10^{ - 3} \;{\text{m}}^{2} /{\text{s}}^{3}$; and $g(t)$ is the following modulation function.

48 $$g(t) = \left\{ {\begin{array}{*{20}c} {(t/t_{1} )^{2} \, 0 \le t \le t_{1} } \\ {1 \, t_{1} \le t \le t_{2} } \\ {e^{{ - a(t - t_{2} )}} \, t_{2} \le t \le t_{3} } \\ \end{array} } \right.$$

with $$t_{1} = 6\;{\text{s}}$$, $$t_{2} = 18\;{\text{s}}$$, $$t_{3} = 30\;{\text{s}}$$ and $$a = 0.18$$.

In this example, the random vibration analysis of the frame structure under non-stationary random excitations is implemented by using the proposed method and the traditional MCS method. The dynamic time-history analysis is carried out based on the Newmark-*β* integration scheme for the traditional MCS method. In addition, the bilinear model on the Midas software platform^34^ is adopted to check the Bouc-wen model used in this study, and the two models are equivalent to each other according the principle of the equal bending moment. The batch function of the Midas software platform is used for the MCS. The above three methods are carried out on the same computer. The time step used for the above methods is set to be $\Delta t{ = }0.01\;{\text{s}}$.The number of samples used in the MCS is $N{ = }2 \times 10^{3}$.

**Figure 4:**
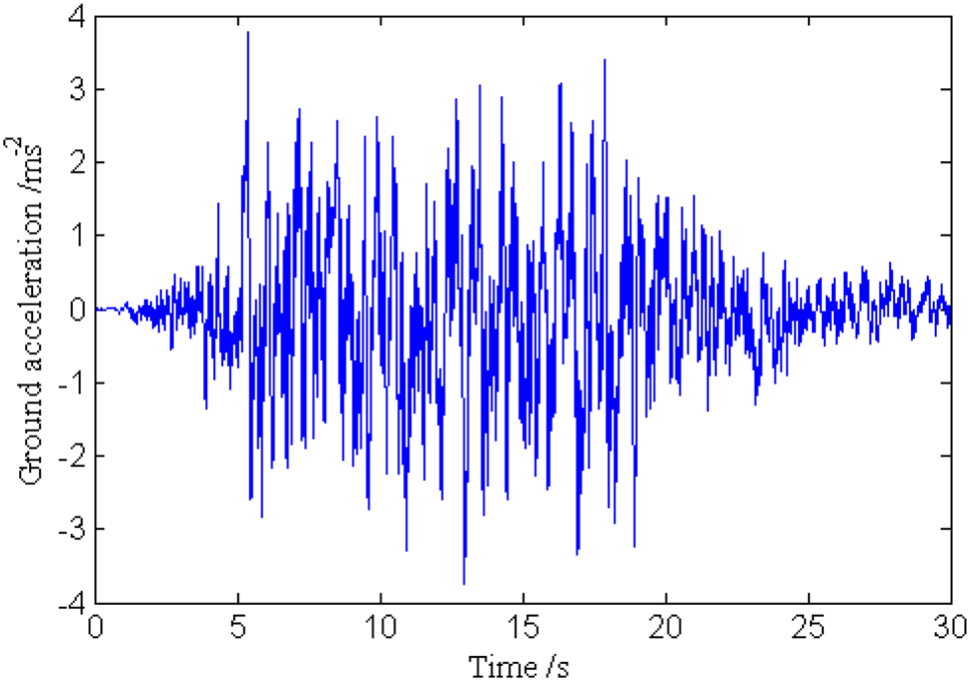
A sample of excitation.

**Figure 5:**
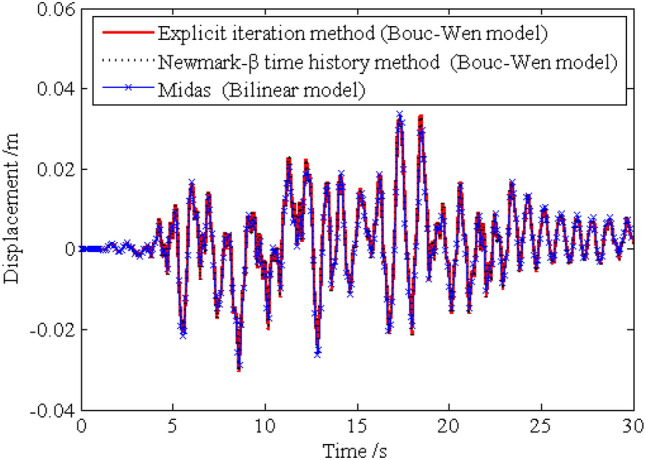
Time history of horizontal displacement of node 16.

**Figure 6:**
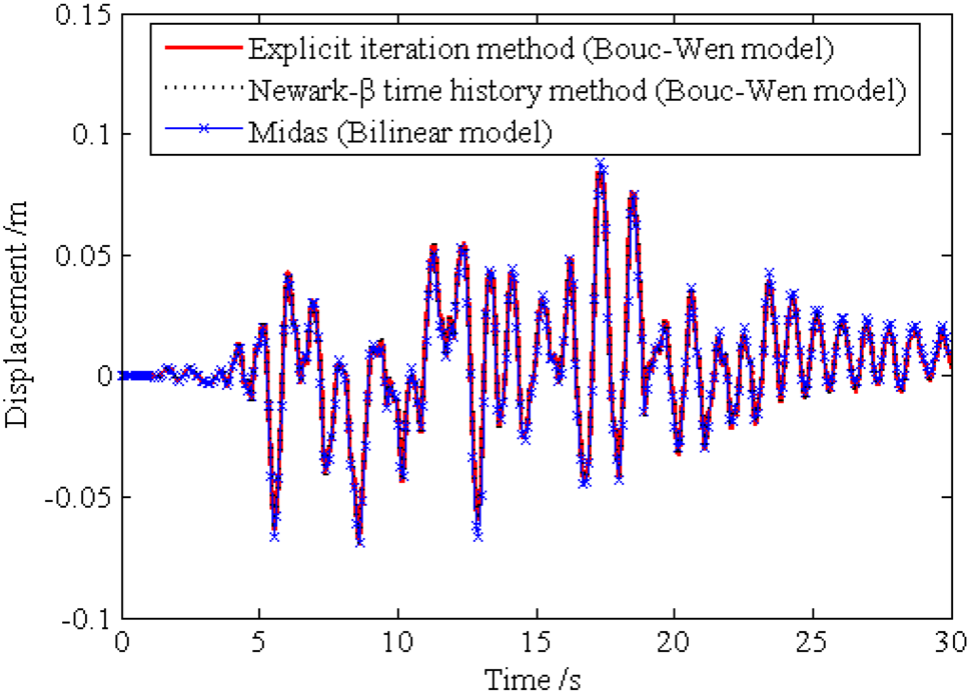
Time history of horizontal displacement of node 28.

**Figure 7:**
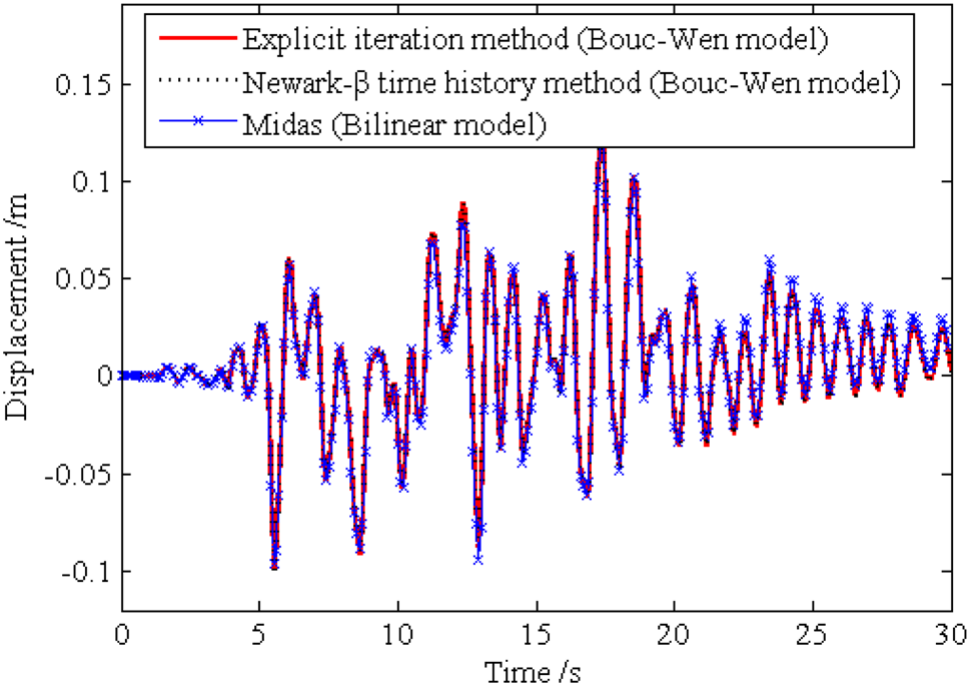
Time history of horizontal displacement of node 40.

**Figure 8:**
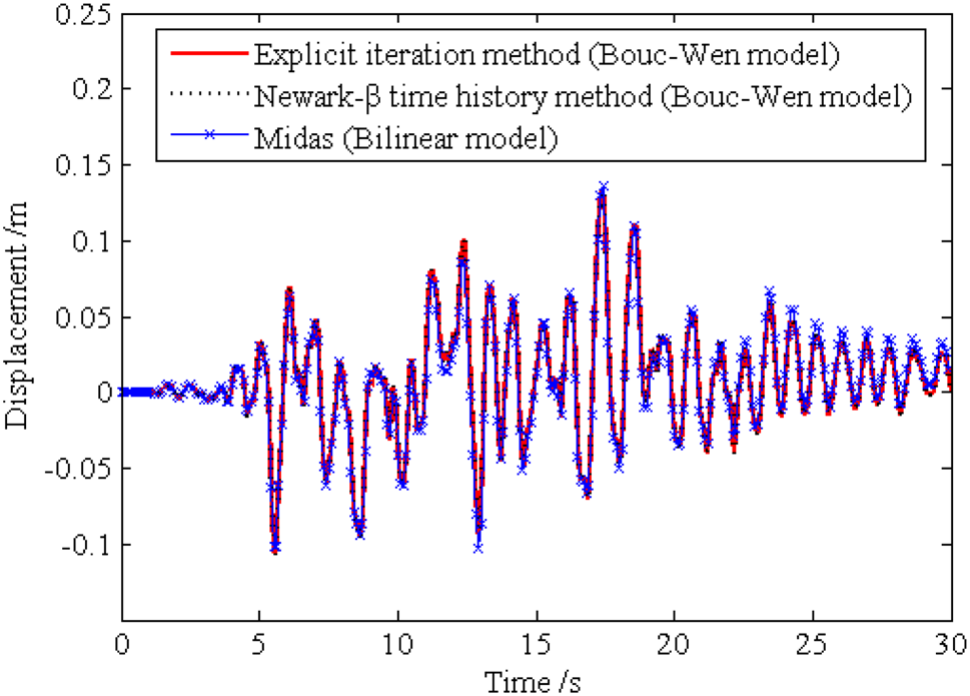
Time history of horizontal displacement of node 52.

**Figure 9:**
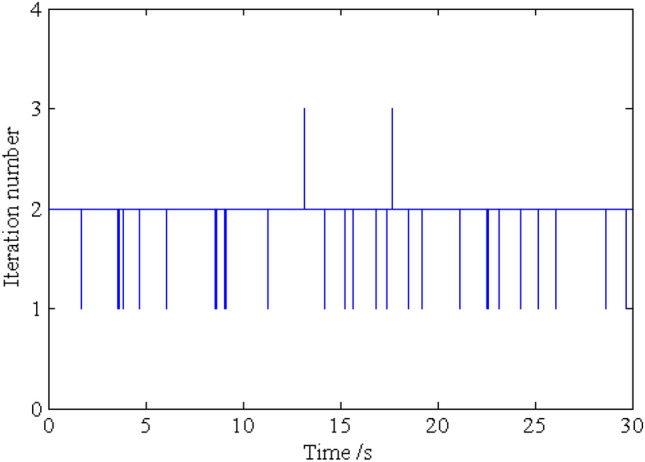
Number of outer iteration for each time step of the explicit iteration method.

In order to investigate the solution precision of the dynamic responses obtained by the proposed method, the deterministic dynamic analysis is carried out based on the above methods. One of the excitation samples is shown in Fig. 4. The horizontal displacements of nodes 16, 28, 40 and 52 of the frame structure under this excitation sample are shown in Figs. 5 ,6 , 7, 8, respectively. It can be seen from Figs. 5–8 that the results of the explicit iteration method and the traditional Newmark-*β* integration scheme are in good agreement. In addition, the results obtained by the Midas software platform agree well with the results of the other two methods, further indicating that the results of the other two methods are correct. The number of outer iterations for each time step of the explicit iteration method under this excitation sample is shown in Fig. 9. As shown in Fig. 9, the numbers of iterations are no more than 3 times, indicating fast convergence rate of the proposed method. The time consumed by the explicit iteration method and the traditional Newmark-*β* integration scheme is 1.973 s and 40.611 s, respectively, for which the latter is 20.6 times of the former (as shown in Table 1). That’s because it needs to update the stiffness matrix and calculate its inverse repeatedly for the traditional Newmark-*β* integration scheme.

**Table 1:** Comparison of the elapsed time (Number of DOF* n* = 144).

Method	A single sample analysis		Random analysis			
			*N* = 2×10^3^		*N* = 1×10^4^	
	CPU time (s)	*T*_2_/*T*_1_	CPU time (s)	$T_{2} {/}T_{1}$	CPU time (s)	$T_{2} {/}T_{1}$
Proposed method (*T*_1_)	1.973	–	3.812 × 10^3^	–	1.896 × 10^4^	–
Traditional method (*T*_2_)	40.611	20.6	8.122 × 10^4^	21.3	4.061 × 10^5^	21.4
Midas (*T*_2_)	21.383	10.8	4.277 × 10^4^	11.2	2.138 × 10^5^	11.3

Note: *N* is the number of samples used in the MCS.

**Figure 10:**
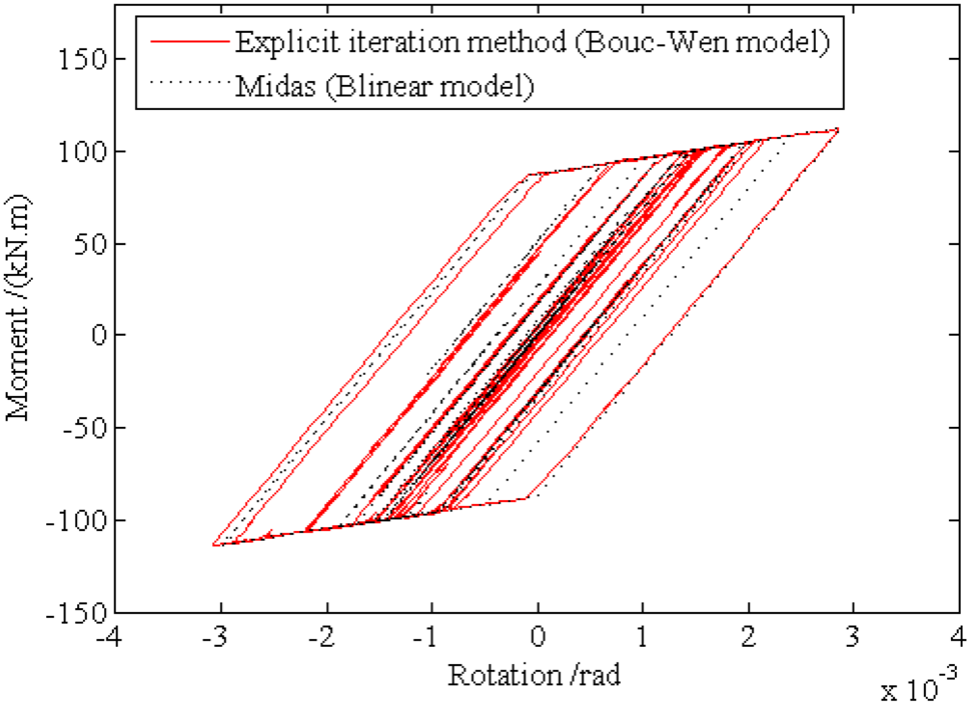
Relation curve of the end rotation and moment of element 1.

**Figure 11:**
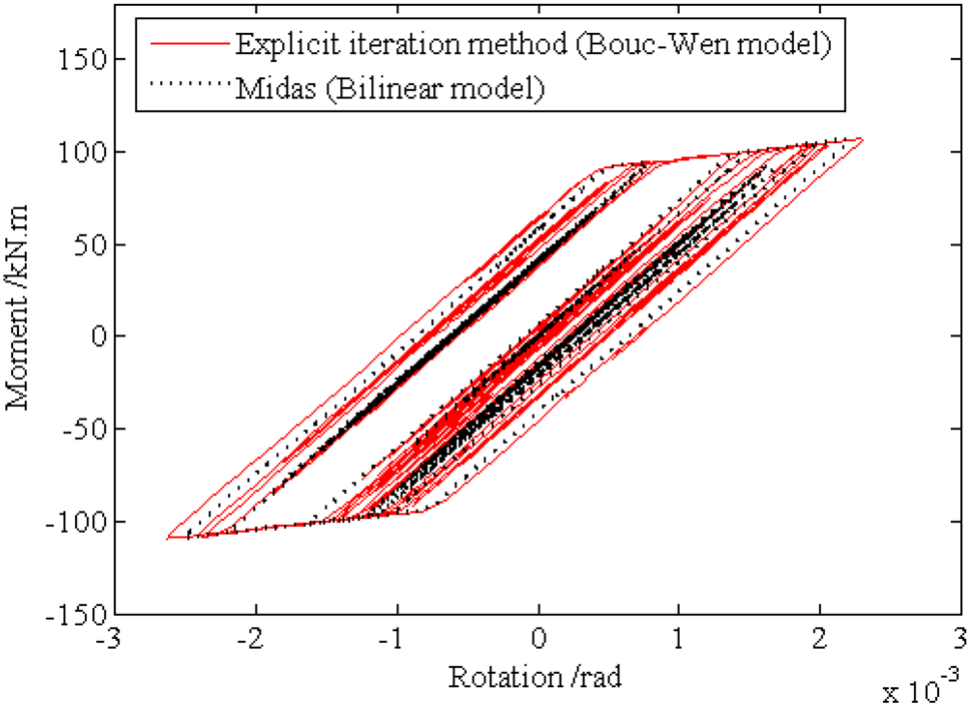
Relation curve of the end rotation and moment of element 2.

**Figure 12:**
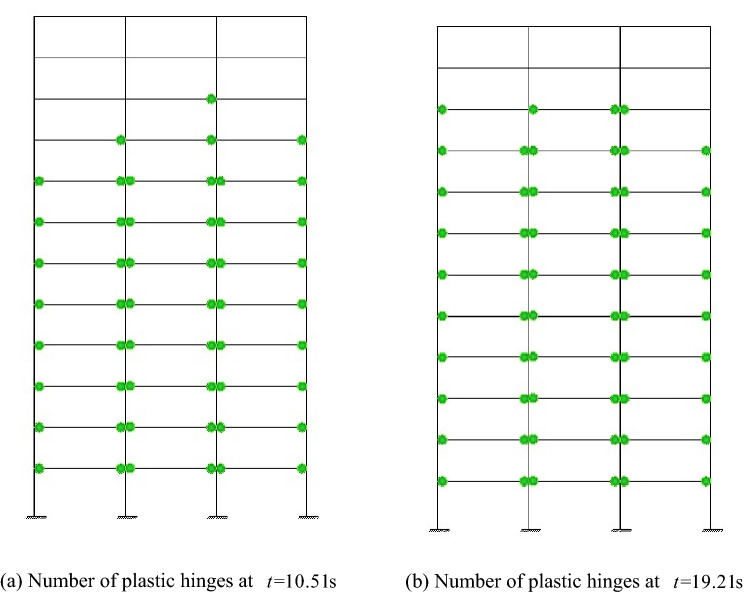
State of plastic hinges at different time instants.

Under the given excitation sample shown by Fig. 4, the relation curves of the rotation and the moment of the left ends for beam 1 and beam 2, which are corresponding to the node $i$ of element $e$ in Fig. 1, are shown in Figs. 10 and 11, respectively, from which it can be seen that the hysteretic curve obtained by the explicit iteration method based on the Bouc-Wen model agrees well with that based on the bilinear model obtained from the Midas software platform, proving the validity of the Bouc-Wen model. Furthermore, the number of plastic hinges occurring at $t{ = }10.51{\text{s}}$ and $t{ = }19.21{\text{s}}$ is shown in Fig. 12. It is worth noting that the beams in the low storeys of the frame structure enter the elastoplastic state earlier than the beams in the upper storeys. Obviously, the beams in the top two storeys nearly keep in linear state through the energy dissipation of the beams in the middle-low storeys.

**Figure 13:**
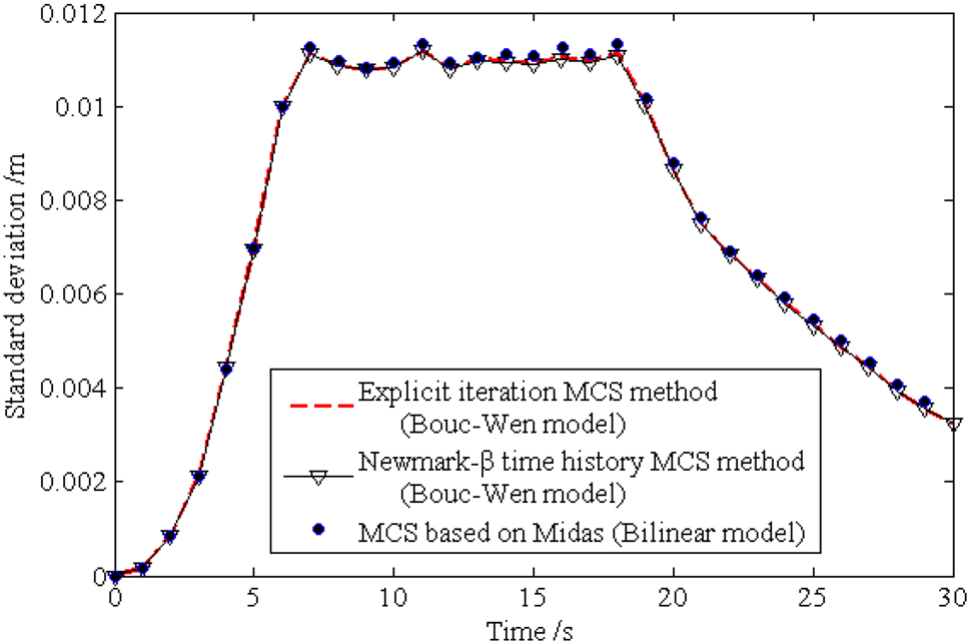
Time history of standard deviation for horizontal displacement of node 16.

**Figure 14:**
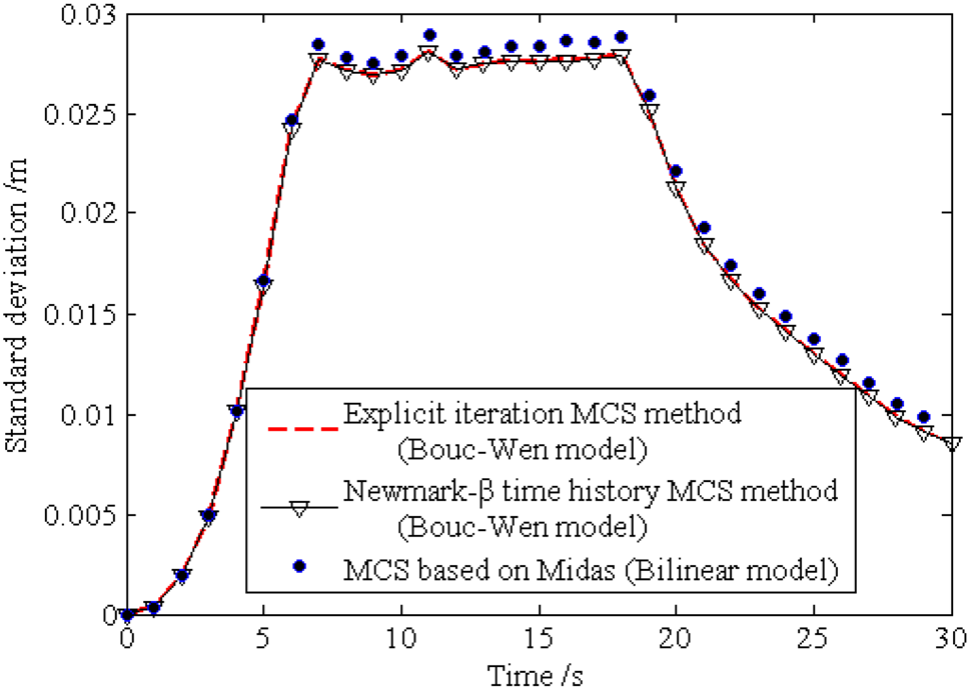
Time history of standard deviation for horizontal displacement of node 28.

**Figure 15:**
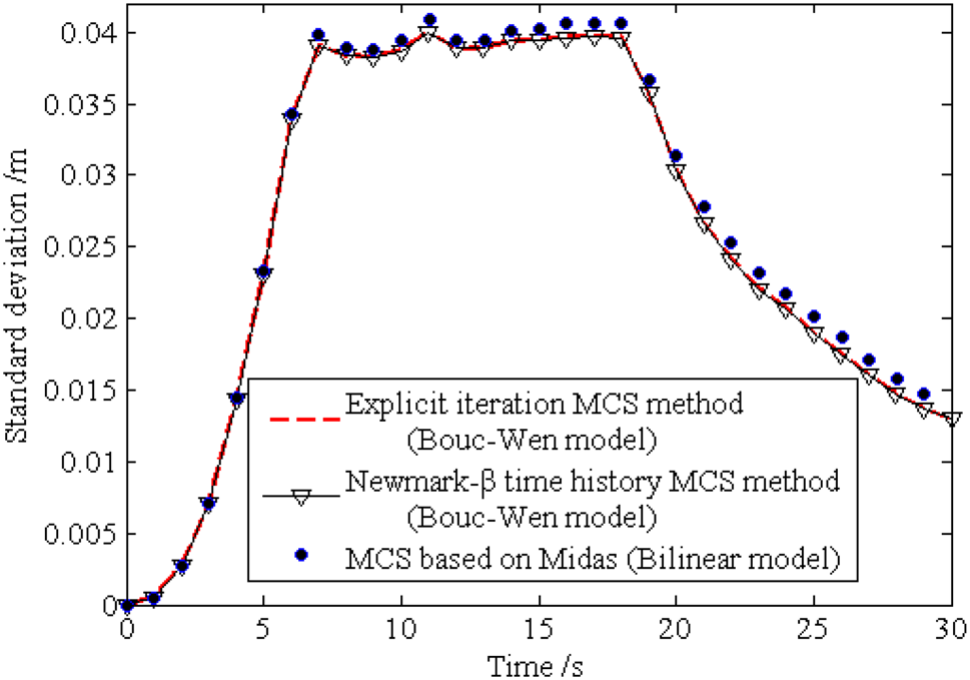
Time history of standard deviation for horizontal displacement of node 40.

**Figure 16:**
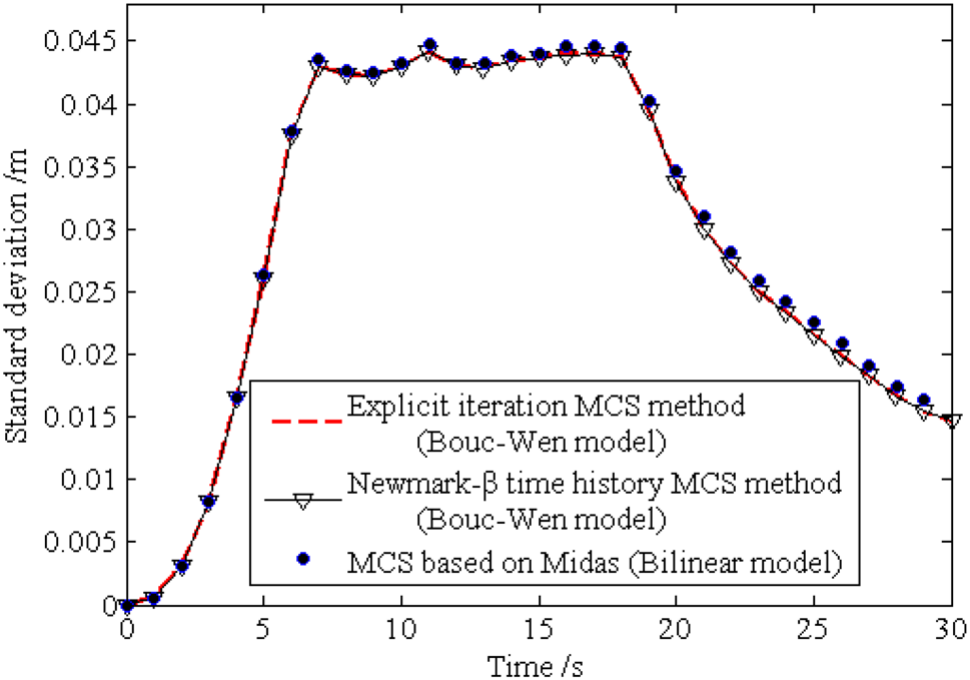
Time history of standard deviation for horizontal displacement of node 52.

**Figure 17:**
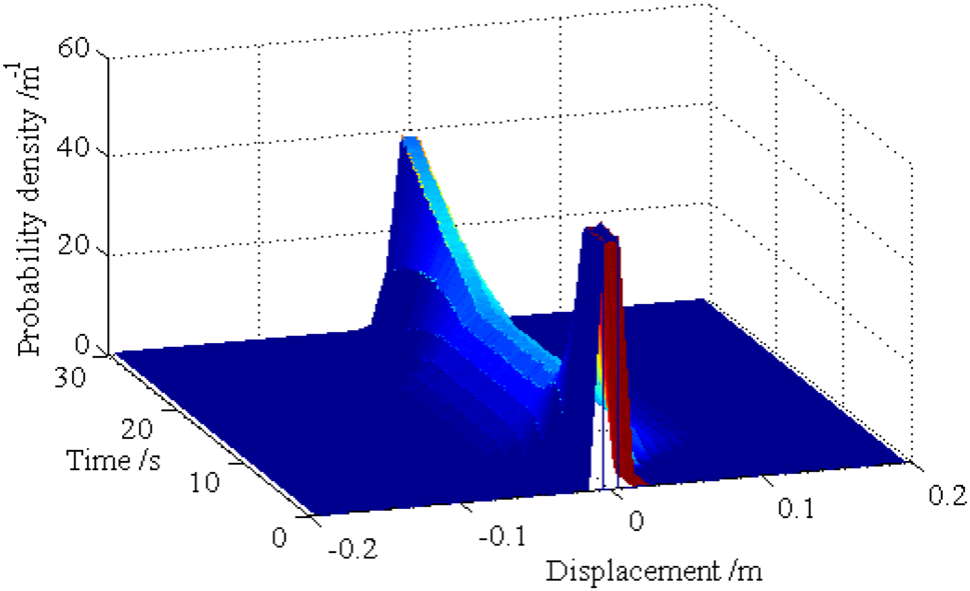
Evolutionary probability density function for horizontal displacement of node 28.

**Figure 18:**
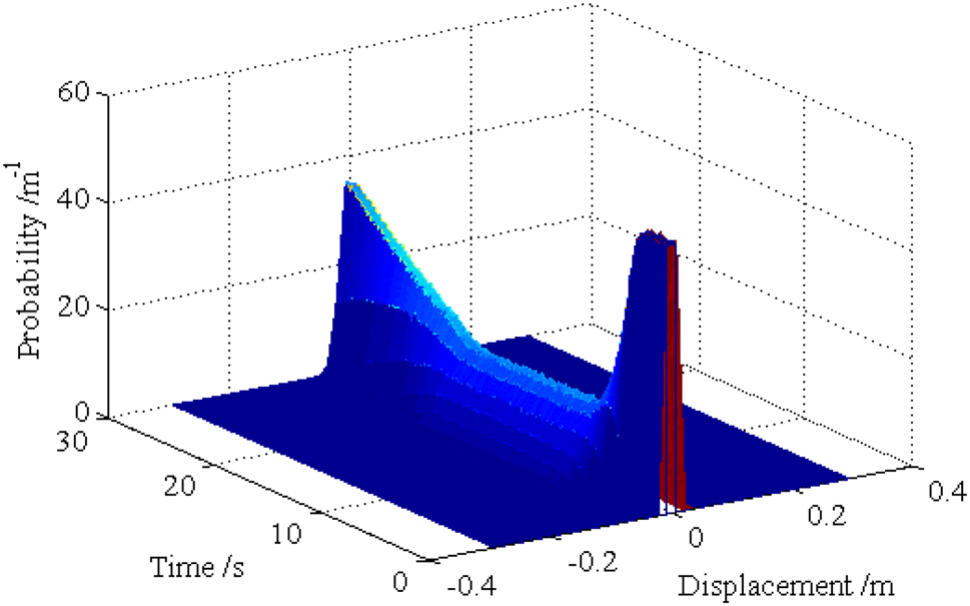
Evolutionary probability density function for horizontal displacement of node 52.

The standard deviations of the horizontal displacements for nodes 16, 28, 40 and 52 are shown in Figs. 13, 14, 15, 16, respectively. As shown by the results, the explicit iteration MCS method and the traditional MCS method have the same accuracy. The results obtained from the Midas software platform also agree well with that obtained by the above two methods. The time elapsed by the above three methods is 3.812 × 10^3^ s, 8.122 × 10^4^ sand $4.277 \times 10^{4} \;{\text{s}}$, respectively, and the comparison of the efficiency for the three methods is shown in Table 1. It indicates the proposed method is still much more efficient than the other two methods for the random analysis once more. In light of the high computational efficiency of the proposed method, the evolutionary probability density functions of the horizontal placements of nodes 28 and 52 can be obtained by increasing the number of excitation sample up to $N{ = }1 \times 10^{4}$, as shown in Figs. 17 and 18. In such a case, the proposed method still shows high computational efficiency, as shown in Table 1.

Lastly, the number of DOFs of the frame structure is increased up to 1200 so as to investigate the computational efficiency of the proposed method. In such a case, the number of excitation samples used is $N{ = }1 \times 10^{3}$. As the computational efficiency of the Midas is superior to the traditional MCS method, the Midas is used to substitute the Newmark-*β* integration scheme for the traditional MCS method in this part. The time elapsed by the proposed method and the Midas is shown in Table 2. As can be seen from Table 2, the proposed method is still high efficient for larger-scale model.

**Table 2:** Comparison of the elapsed time (Number of DOF *n* = 1200).

Method	A single sample analysis		Random analysis (*N* = 2×10^3^)	
	CPU time (s)	*T*_2_/*T*_1_	CPU time (s)	*T*_2_/*T*_1_
Proposed method* T*_1_	25.118	–	$2.401 \times 10^{4}$	–
Midas *T*_2_	247.138	9.8	$2.471 \times 10^{5}$	10.2

Note: *N* is the number of samples used in the MCS.

## Conclusions

This paper proposes a novel approach for analyzing random vibrations in nonlinear frame structures subjected to random seismic excitations. The explicit time-domain method is improved in this approach by integrating the plastic hinge model, which can simulate the nonlinear behaviors caused by material property changes. Specifically, the hysteretic system’s equation of motion is constructed using auxiliary differential equations that govern the plastic rotational displacements and their corresponding hysteretic displacements. Additionally, by introducing the concept of equivalent excitations, an explicit iteration scheme is developed for resolving the equation of the hysteretic system, where the auxiliary differential equations are solved under the assumption that the plastic rotational velocity changes linearly with time between two adjacent time instants. Finally, by combining the Monte Carlo simulation method and the proposed explicit time-domain method, the non-stationary random responses of the nonlinear frame structures can be obtained. Numerical examples are examined and the following conclusions are drawn:

1. The proposed method is an extension of the explicit time-domain method. It enables the performance simulation of engineering structures with plastic hinges subjected to random seismic excitations while maintaining high solution accuracy and computational efficiency. The proposed algorithm can be applied to random nonlinear behavior simulation of frame structures.
2. The proposed iterative solution method with inner and outer loops resolves equations involving displacements describing the frame’s global state, plastic rotational displacements, and corresponding hysteretic parameters. This method produces a novel concept for solving problems involving nonlinear coupled variables of multiple types. Based on this novel concept, more effective iterative algorithms for nonlinear simulation of structures with additional physical and model parameters are expected to be developed, which will contribute to the development in the field of engineering numerical calculation.

## Data Availability

The datasets used and/or analysed during the current study available from the corresponding author on reasonable request.
